# Minimally access versus conventional hydrocelectomy: a randomized trial

**DOI:** 10.1590/S1677-5538.IBJU.2014.0248

**Published:** 2015

**Authors:** Aly Saber

**Affiliations:** 1 Department of General Surgery, Port-Fouad general Hospital, Port-Fouad, Egypt

**Keywords:** Minimally Invasive Surgical Procedures, complications [Subheading], Testicular Hydrocele

## Abstract

**Objective:**

To compare our previously published new minimally access hydrocelectomy versus Jaboulay's procedure regarding operative outcome and patient's satisfaction.

**Materials and Methods:**

A total of 124 adult patients were divided into two groups: A and B. Group A patients were subjected to conventional surgical hydrocelectomy (Jaboulay's procedure) and group B patients were subjected to the new minimal access hydrocelectomy. The primary endpoint of the study was recurrence defined as a clinically detectable characteristic swelling in the scrotum and diagnosed by the two surgeons and confirmed by ultrasound imaging study. The secondary endpoints were postoperative hematoma, wound sepsis and persistent edema and hardening.

**Results:**

The mean operative time in group B was 15.1±4.24 minutes and in group A was 32.5±4.76 minutes (P≤0.02). The mean time to return to work was 8.5±2.1 (7–10) days in group B while in group A was 12.5±3.53 (10–15) days (P=0.0001). The overall complication rate in group B was 12.88% and in group A was 37%. The parameters of the study were postoperative hematoma, degree of scrotal edema, wound infection, patients’ satisfaction and recurrence.

**Conclusion:**

Hydrocelectomy is considered the gold standard technique for the treatment of hydrocele and the minimally access maneuvers provide the best operative outcomes regarding scrotal edema and hardening and patient's satisfaction when compared to conventional eversion-excision hydrocelectomies.

## INTRODUCTION

Hydrocele is the most common benign scrotal swelling with estimated incidence as one percent of the adult male population (1). A controversy exists about the treatment of primary vaginal hydrocele. Aspiration and sclerotherapy have been described; however hydrocelectomy remains the treatment of choice for the management of hydroceles (2). Aspiration and sclerotherapy with doxycycline seems as effective and safe as nonsurgical treatment option for hydrocele where the success rate of a single hydrocele aspiration and sclerotherapy procedure is claimed to have the same success rates involving hydrocelectomy while avoiding the hospital expense and many other complications (3); other studies reported lower success rate and less patient's satisfaction than hydrocelectomy (4, 5). Hydrocelectomy through eversion procedures for hydrocele may cause postoperative discomfort, temporary limitation of normal activities and complications, such as hematoma, infection, persistent swelling, chronic pain and decreased fertility (6, 7). The author in the present study compared his previously published new minimally access hydrocelectomy (8) versus Jaboulay's procedure (9).

### Objectives

The aim of this prospective randomized study was to compare the author's previously published new minimally access hydrocelectomy versus Jaboulay's eversion procedure in adult patients regarding operative outcome and patient's satisfaction.

## PATIENTS AND METHODS

### 

#### Patients

A total of 124 adult patients, aged 18-56 years within the period April 2006 to October 2011, with diagnosis of hydrocele were enrolled to this prospective randomized study and divided into two equal groups A and B. Group A (N=62) patients were submitted to conventional surgical hydrocelectomy (Jaboulay's procedure) while group B patients (N=62) were submitted to the new minimal access hydrocelectomy. All patients were subjected to either conventional eversion or the new minimal access hydrocelectomy as an ambulatory procedure with general anesthesia or spinal analgesia. The cohort of our patients represents adult males working in Port-Said free industrial zone, Port-Said, Egypt. Patients came from more than 4 governorates; Port-Said, Ismailia, Sharkya and Kafr el-Sheikh.

Written consents were obtained from all patients before the study. The steps of both operative interferences were explained to all patients. The local ethics committee had approved all operative procedures. Ethical approval for this study was granted by the ethical review committee under supervision of the general director of Port-Fouad general hospital, Port-Fouad, Port-Said, Egypt.

#### Sample size

In general, the overall complications rate of conventional surgical hydrocelectomy in previous studies is about 40% (5) and those of minimally access hydrocelectomy is about 14% (8). Calculation of the sample size included the number of participants to be recruited for the study using the mathematical equation. The authors used these two equations to calculate the minimum number required to reliably answer the research question. Using the first equation (10), the number, N=62 patients for each group, was given by:


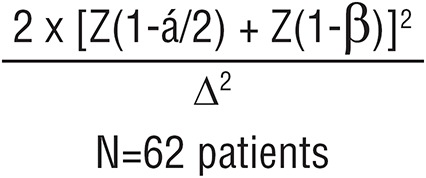


where z (1-a/2) and z (1-β) represent percentage points of the normal distribution for statistical significance level (ά) at 0.05 value is 1.96 and power (1-β) with accepted 95% positive rate is 1.6449, where β, the false-negative rate. Δ represents the standardized difference (i.e. the treatment difference divided by its standard deviation):


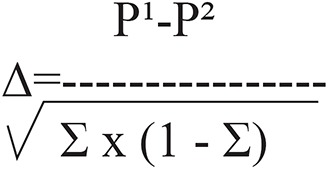


Standardized difference


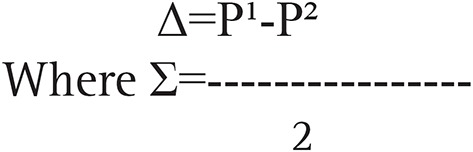


P¹ represents the overall complications of conventional surgical hydrocelectomy in previous studies=40% (5).

P² represents the overall complications of minimally access hydrocelectomy reported in previous studies=17% (8).

#### Randomization

Randomization was performed prior to study commencement as follows: Opaque envelopes were numbered sequentially from 1 to 124. A computer-generated Table of random numbers was used for group assignment; if the last digit of the random number was from 0 to 4, assignment was to Group A (conventional surgical hydrocelectomy), and if the last digit was from 5 to 9, assignment was to Group B (minimally access hydrocelectomy). The assignments were then placed into the opaque envelopes and the envelopes sealed. As eligible participants were entered into the trial, these envelopes were opened in sequential order to give each patient his random group assignment. The envelopes were opened by the operating surgeon after patient consent and just prior to the surgery (10).

#### Preoperative workup

The detailed history and full physical examination of each patient were assessed. The diagnosis was confirmed by fluctuation and trans-illumination. Laboratory investigations like hemoglobin, white blood cell count and urine routine examination were done in all. Scrotal ultrasound imaging was done in all patients.

#### The surgical techniques

Cephradine 1gm IV at the time of induction of anesthesia or just after the administration of spinal anesthesia was given followed by another dose 2 h postoperatively. Patients were seen in the second day and examined for scrotal edema and hematoma. In all of group B patients, drains were removed in the second day while in those of group A drains were removed in the third day. All the excised tissues were sent for pathological examination to rule out any epididymal or vasal structures in the specimen.

Jaboulay's procedure: The testis was delivered through an incision in the scrotum, the tunica was opened and everted and most of the hydrocele sac was resected with electrocautery, leaving a reasonable cuff along the borders of the testicle. Bleeding was controlled by a running suture closing the free edges of the hydrocele sac and hemostasis was secured by the aid of electrocautery. Standard two-layer closure was used to close the scrotum with small tube drain (9).

The new minimally access hydrocelectomy: A small scrotal incision 2cm long was done and incision of the Dartos muscles in the same line was done with electrocautery (Figures 1A and B). The parietal tunica vaginalis (PTV) was grasped and minimal blunt dissection was made by the aid of the index finger and a small hole was made for aspiration of hydrocele fluid (Figure-2A). Then a disc of tissue was excised of the PTV about double of the skin incision dimension using electrocautery. The edge of the visceral surface tunica vaginalis was sutured to the parietal layer of the tunica vaginalis and then to the Dartos (Figure-2B) and all were sutured to scrotal skin in an everted manner aiming to expose the visceral tunica toward scrotal skin (Figures 3A and B). If the visceral surface of the tunica vaginalis is sutured to the Dartos, eversion will be created. Then when this everted structure is sutured to the scrotal skin, it put in contact the sac with lymph-rich subcutaneous tissues. A drain was left in place and discharge was allowed at the same day (8).

**Figure 1A:**
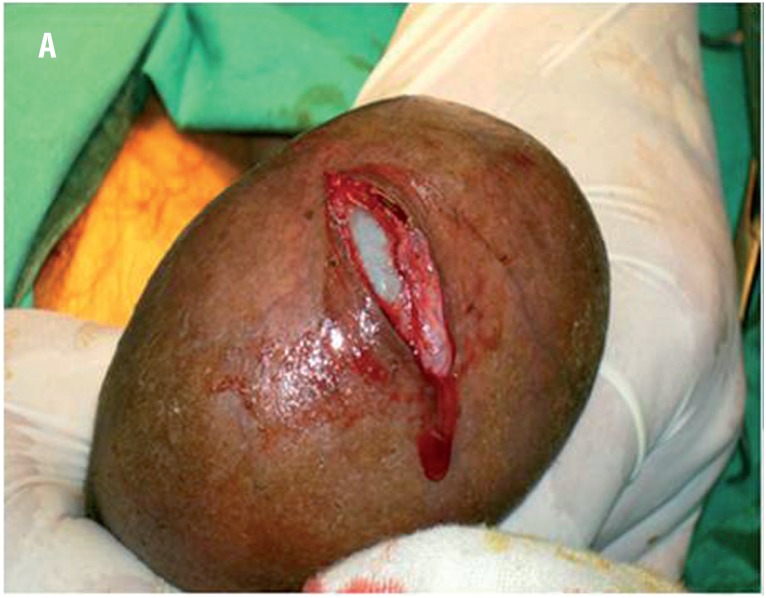
An operative photograph showing the length of the scrotal skin incision, 2cm (it appears longer due to stretch of the skin by the assistant).

**Figure 1B:**
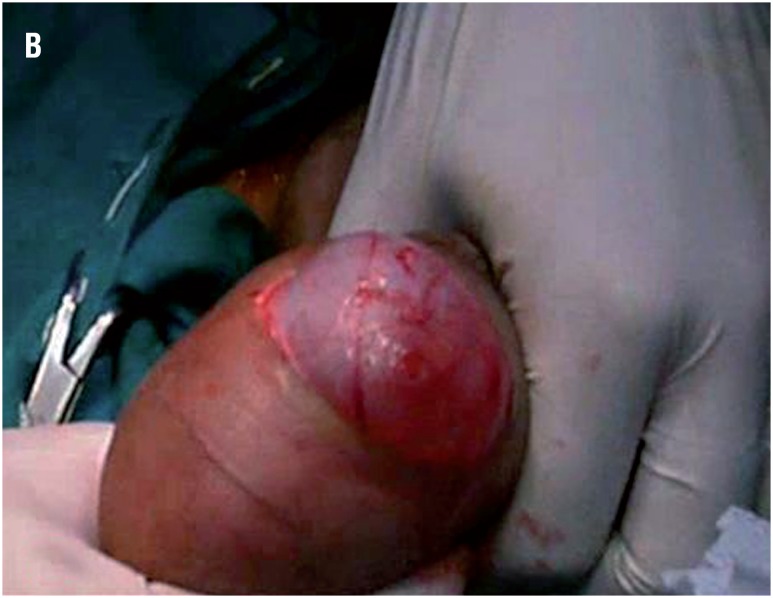
An operative photograph showing delivery of the hydrocele sac through the small scrotal skin.

**Figure 2A:**
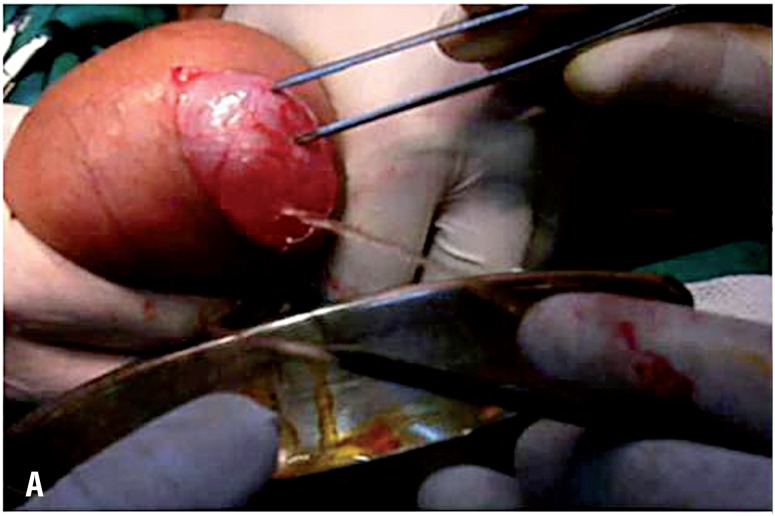
An operative photograph showing evacuation of hydrocele fluid through a small hole made in the tunica vaginalis.

**Figure 2B:**
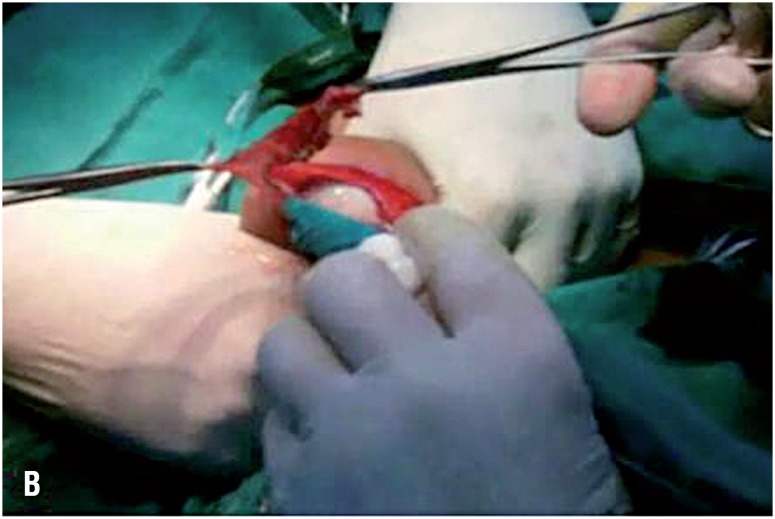
An operative photograph showing in situ excision of the hydrocele sac using electrocautery.

**Figure 3A:**
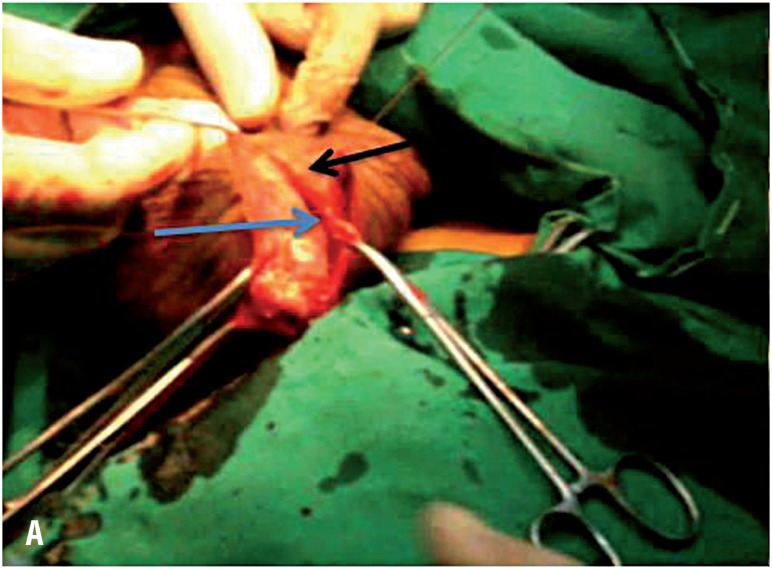
An operative photograph showing the beginning of the eversion technique. Blue arrow points to the visceral tunica vaginalis while the black one points to the parietal tunica.

**Figure 3B:**
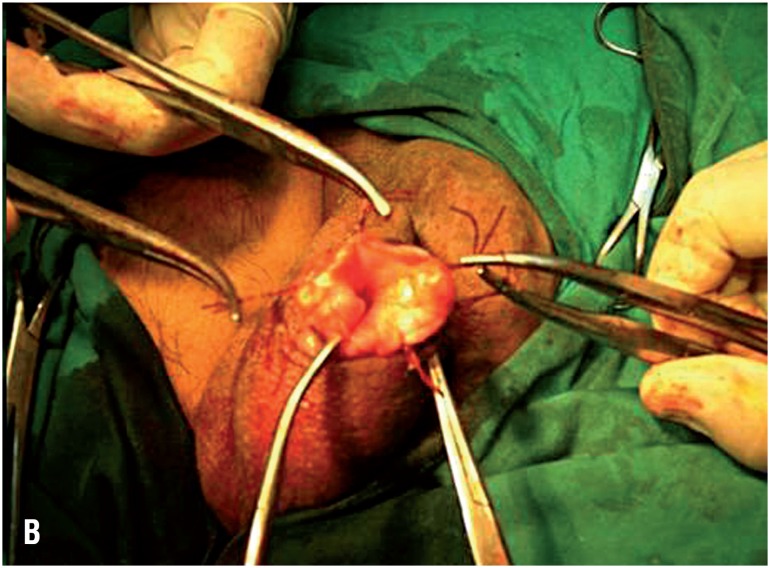
An operative photograph showing completed eversion technique by suturing of the edge of the tunica to the Dartos and scrotal skin in an everted manner aiming to expose the visceral tunica toward scrotal skin.

#### End Points

The primary endpoint of the study was recurrence defined as a clinically detectable characteristic swelling in the scrotum and diagnosed by the two surgeons and confirmed by ultrasound imaging study. The secondary endpoints were postoperative hematoma, wound sepsis and persistent edema and hardening.

### Statistical analysis

Data were entered and analyzed using SPSS (Statistical Package for Social Sciences) software program version 15.0 for analysis. Values were expressed as means±standard errors of deviation. Student t test was used to compare categorical variables. P value set at <0.05 for significant results.

## RESULTS

There was no statistical significant difference between the two groups regarding age, body mass index, duration of symptoms and size of hydroceles. Age ranged between 18–56 years with a mean age of 37±11.4 years. Follow-up included patients’ complaint, if any, clinical examination and ultrasonography. The maximum follow-up period was 96 months and the minimum was 28 months with a mean value of 59.88±24.22 months. None of our patients missed their follow-up program because of the obligatory visit designed by the health organization of the company they work for.

The operative time in group B ranged between 12-18 minutes and the mean was 15.1±4.24 minutes and in group A ranged between 25-40 minutes with mean value 32.5±4.76 minutes with significant distribution (P≤0.02). The mean time of hospital stay for group B was 13.48±6.38 hours with 10 hours as a minimum and 30 hours as a maximum value, while in group A was 21.19±11.65 hours with 12 hours as a minimum and 48 hours as a maximum value but this distribution was not significant (P≥0.05). Time off from work was defined as the number of days between the day of surgery and the first day a patient returned to work (10). The mean time to return to work was 8.5±2.1 (7–10) days in group B while in group A was 12.5±3.53 (10–15) days. The mean time off from work in group B was 9±2.35 days and in group A was 13.5±4. (P=0.0001).(Table-1).

**Table 1:** Mean operative time, hospital stay and time off from work in both groups.

Item	Group A	Group B	P value
Operative time (minutes)	32.5±4.76	15.1±4.24	≤0.02
Hospital stay (hours)	21.19±11.65	13.48±6.38	≥0.05
Time off from work (days)	13.5±4.	9±2.35	=0.0001

We relied on the previously reported data regarding our new minimally invasive hydrocelectomy (8) for the common postoperative findings: postoperative hematoma, degree of scrotal edema, wound infection, patients’ satisfaction and recurrence. The overall complication rate in group B was 12.88% and in group A was 37%.

Postoperative hematoma was not observed in any of our patients in group B while mild hematoma treated by conservative measures was detected in three patients in group A (4.8%). Mild and moderate scrotal edema usually subsided within a few days postoperatively (7) while scrotal edema and hardening was considered when pain and swelling interfered with daily activities (11). The present study showed that scrotal edema was inevitable as we observed that mild and moderate scrotal edema and hardening occurred in all patients of both groups with varying proportions. In group A, scrotal edema and hardening represented the higher incidence while mild degree was the least form of scrotal edema. In contrast, in group B mild and moderate degrees formed the majority of patients and scrotal edema and hardening occurred only in three patients (P≤0.05). Persistent edema and hardening were confined to the ipsilateral hemiscrotum and required additional bed rest and anti-inflammatory agents. Mild to moderate cellulitis was seen in four patients in both groups A and B (6.45%). All our patients of group B were completely satisfied with this new minimally invasive procedure by the end of second postoperative week and all over the follow-up periods and only three patients (4.83%) were un-satisfied due to scrotal hardening while in group A, scrotal edema and hardening was observed in 24.2%. Disease recurrence was confirmed by two treating surgeons and by the aid of US study. There was disease recurrence in one patient (1.6%) in both groups A and B (Table-2).

**Table 2:** Overall complication rate and patient's satisfaction in both groups.

Item	Group A	Group B	P value
Overall complications	37%	12.88%	P≤0.05
Postoperative hematoma	3 (4.8%).	–	NS
Edema & hardening	15 (24.2%)	3 (4.8%).	P≤0.05
Wound sepsis	4 (6.45%)	4 (6.45%)	NS
Patient's satisfaction	95.2%	75.8%	P≤0.05
Recurrence	1 (1.6%)	1 (1.6%)	NS

**NS** = nonsignificant.

## DISCUSSION

Hydrocelectomy is considered the gold standard technique for the treatment of hydrocele; aspiration and sclerotherapy have fewer complications and the success rate and patient's satisfaction are inferior to hydrocelectomy (8). Jaboulay's procedure for hydrocelectomy has satisfactory rate of success but with less patient satisfaction due to postoperative scrotal hardening (6, 9, 12). Minimally access hydrocelectomy was performed through fenestration of the tunica (8) and pull-through technique to remove large hydrocele sacs through a small incision and with minimal dissection (7). Our new minimally access technique of hydrocelectomy was previously published (8). The hydrocele sac is treated through smaller scrotal skin incision together with excision of smaller disc of the hydrocele sac.

The mean operative time in our study was longer in group A than group B with statistical significant distribution because much time was lost in partial excision of the sac as well as to achieve haemostasis (6, 9, 12). Also, the time off from work was longer for patients of group A than group B with statistical significant distribution and this came in concordance with published data of same interest (4, 13).

The overall complication rate in group B was lower than in group A and came in agreement with other reports of previous studies of same interest (5–8). The overall incidence of postoperative complications is significantly lower among patients with less operative trauma (14). In less invasive techniques (7, 8) as well as our minimally invasive maneuver, the overall complications rate was inferior to those in eversion and excision hydrocelectomy (5, 6, 15).

Postoperative haematoma and scrotal edema and hardening were observed with higher incidence in group A patients where there was more tissue dissection than those in group B. Excision––eversion technique invites edema and hematoma due excessive handling and wide dissection of the hydrocele sac (5–7). In the current technique a disc of the hydrocele sac is pulled and resected through a small scrotal incision with minimal dissection. So, the hematoma formation was not seen in group B patients as well as patients of the in situ techniques (6) while in conventional hydrocelectomy, the hematoma formation may reach up to 3.3% (4, 14).

In scrotal surgery, significant postoperative infection occurred in patients subjected to more operative trauma and ranged from superficial surgical site infection, scrotal abscess formation and pyocele (5, 7, 8) with a rate of incidence between 5-14% (16). In the present study, superficial surgical site infection confined to the scrotal skin as mild to moderate cellulitis was detected in four patients in both groups that needed additional course of Cephradine orally (1gm 12/12hs).

Regarding recurrence, there was disease recurrence in one patient (1.6%) in both groups A and B, while others reported 1.3-7% recurrence (8, 17).

The assessment of the patient's satisfaction with the treatment procedure depends on physician's instructions for postoperative period, the postoperative outcome, the follow-up period and the success rate (18). Regarding the success rate, it was reported that the level of satisfaction was superior with hydrocelectomy when compared with sclerotherapy due to fewer incidence of recurrence (4).

The most common complications following scrotal surgery for hydrocele are persistent scrotal swelling and hardening (6) while with minimally access procedures (7, 8) as in the present study, the scrotal swelling and hardening are much less than the eversion-excision hydrocelectomy (6–8, 14) A total of 95.17% of our patients of group B were completely satisfied with this new minimally invasive procedure by the end of the second postoperative week and all over the follow-up periods and only three patients (4.83%) were unsatisfied due to scrotal hardening. In group A, 24.2% of patients were unsatisfied.

## CONCLUSIONS

Hydrocelectomy is considered the gold standard technique for the treatment of hydrocele and the minimally access maneuvers provide the best operative outcome regarding scrotal edema and hardening and patient's satisfaction when compared to conventional eversion-excision hydrocelectomies.
